# Metformin in combination with JS-K inhibits growth of renal cell carcinoma cells via reactive oxygen species activation and inducing DNA breaks

**DOI:** 10.7150/jca.36372

**Published:** 2020-03-31

**Authors:** Yuwan Zhao, Qiuming Luo, Jierong Mo, Jianwei Li, Dongcai Ye, Zhixian Ao, Lixin Chen, Jianjun Liu

**Affiliations:** Laboratory of Urology, Affiliated Hospital of Guangdong Medical University, Zhanjiang, Guangdong 524001, China

**Keywords:** metformin, JS-K, renal cell carcinoma, ROS, DNA breaks

## Abstract

Metformin (MET) is taken as a principal medication for remedying Type 2 diabetes mellitus. Its anti-tumor effect has been reported increasingly, but the precise mechanism of it remains unclear. This study aims to explore the efficacy of MET and MET combined with nitric oxide donor prodrug JS-K on the proliferation, apoptosis, and DNA damage in human renal cell carcinoma (RCC) cells, and investigate the possible molecular mechanism involved. The cell proliferation was tested through methyl-tetrazolium assay and cell apoptosis was ascertained by flow cytometry. The dihydroethidium and JC-1 fluorescent methods were used to detect Reactive oxygen species (ROS) and mitochondrial transmembrane potential (Δψm), respectively. Proteins associated with apoptosis and DNA damage were evaluated by Western blotting. Results showed that MET and JS-K could suppress cell growth, and the inhibition concentration 50 of treatment with MET combined with JS-K (MET + JS-K) showed more toxicity than individual agents on RCC cells. This augmented toxicity was associated with intracellular reactive oxygen species (ROS) level, mitochondrial membrane potential alteration, and induced DNA breaks. The results of Western blotting showed that the expression level of pro-apoptotic proteins, such as Bax, Bak, caspase-3, and caspase-9, was up-regulated, and the anti-apoptotic protein Bcl-2 was down-regulated after treatment using MET alone and MET + JS-K, correspondingly. Moreover, MET + JS-K inhibited the expression of cellular PCNA and Rad51, and immunofluorescence analysis of γH2AX proved that MET + JS-K enhanced DNA damage. In summary, the results of this research indicated that MET and JS-K inhibited RCC cell growth by activating ROS, targeting mitochondria-dependent apoptotic pathways, and inducing DNA breaks.

## Introduction

Renal cell carcinoma (RCC) is a frequent progressive cancer of urologic system with poor prognosis and accounts for 3% of adult malignancies 1, 2. Currently, the approved therapy for patients with early partial RCC is radical nephrectomy 3. However, approximately 25%-30% of people who suffer from RCC have obvious metastatic or progressive disease, and the 5-year survival rate is very low (only 10%-12%) 2. Radiotherapy and chemotherapy are treatment options that can be selected by patients with advanced RCC who missed opportunities for surgical treatment 4. However, according to the clinical experience, RCC is extremely insensitive to radiotherapy and conventional chemotherapy drugs 5, 6. Therefore, new drugs with anti-renal cancer effect must be discovered.

MET is a first-line drug for treating Type 2 diabetes. MET increases the absorption and utilization of glucose by skeletal muscles, reduces the insulin resistance of peripheral tissues, and inhibits gluconeogenesis (the production of glucose) in the liver, thereby reducing the blood glucose level 7-10. In 2005, a retrospective analysis by Evans 11 found that the tumor incidence decreased in Type 2 diabetes patients who took MET, thereby indicating the potential anti-tumor effects of MET. In recent years, MET has attracted increasing attention given its evident anti-tumor effect. It mainly inhibits the growth, invasion, and metastasis of virulent tumors like breast cancer, lung cancer, esophageal cancer, nasopharyngeal cancer, and melanoma, via suppressing cell proliferation, prompting cell apoptosis, and blocking cell cycle 12-17.

Nitric oxide (NO) is a lipophilic signaling molecule that is easy to diffuse and has a short biological half-life. NO donor drugs, such as JS-K (C13H16N6O8, CAS-No., 205432-12-8), which was developed by the US National Cancer Institute (NCI), has been reported to be extremely cytotoxic to many kinds of human cancer cells but has no obvious toxicity to normal human cells 18-22.

The emergence of γH2AX is one of the earliest cellular responses to DNA double strand breakage, therefore, γH2AX expression was positively correlated with the degree of DNA damage. PCNA is closed connected with DNA replication and replication-associated processes, while Rad51 plays a critical role in DNA replication and homologous recombination repair, to some extent, the expression of PCNA and Rad51 can reflect the repair level of DNA 23-25.

In this research, we explored the effects of MET and MET combined with JS-K (MET + JS-K) *in vitro* on proliferation, apoptosis, and DNA damage of RCC cell lines (A498 and ACHN). Our findings demonstrate that MET and JS-K inhibit RCC cell growth by activating reactive oxygen species (ROS) and inducing DNA breaks.

## Materials and Methods

### Cell culture

Human RCC cells (A498 and ACHN) and the normal renal cell line (HK-2) were obtained from Guangzhou Jennio Biological Technology Co., Ltd. (Guangzhou, China). A498 cells were grown in RPMI 1640 medium (GIBCO, Thermo Fisher Scientific, Inc., Waltham, MA, USA). ACHN and HK-2 cells were cultured in Dulbecco's modified Eagle medium (DMEM) (GIBCO, Thermo Fisher Scientific, Inc., Waltham, MA, USA). All culture media were supplemented with 10% (v/v) fetal bovine serum (FBS; GIBCO, Thermo Fisher Scientific, Inc., Waltham, MA, USA) at 37°C in a humidified atmosphere that contained 5% CO_2_. The conventional digestion was performed when cell confluence reached 80%-90%, and the media were refreshed every 2 or 3 days.

### Reagents and antibodies

MET was purchased from Beijing Solarbio Science & Technology Co., Ltd. (Beijing, China) and dissolved in phosphate-buffered saline (PBS) as a stock solution of 2 M. The NO prodrug JS-K was purchased from Santa Cruz Biotechnology, Inc. (Dallas, TX, USA) and dissolved in dimethyl sulfoxide (DMSO) as a stock solution of 5 mM. N-acetylcysteine (NAC) and glutathione disulfide (GSSG) were obtained from Beyotime Institute of Biotechnology (Shanghai, China) and dissolved in PBS to concentrations of 100 mM and 5 mM respectively. All stock solutions were stored at -20°C for further use. Antibodies against Bak, Bcl-2-associated X protein, B-cell lymphoma 2, caspase-3, caspase-9, cytochrome *c* (Cyto-C), Phosphorylated histone H2AX (γH2AX), DNA repair protein Rad51, and Proliferating cell nuclear antigen (PCNA) were obtained from Cell Signaling Technology, Inc. (Danvers, MA, USA), and antibody against GAPDH was purchased from Abcam (Cambridge, UK). Horseradish peroxidase-conjugated IgG secondary antibodies were purchased from EarthOx Life Sciences (Millbrae, CA, USA).

### Cell viability assay

Cell viability was assessed by methyl-tetrazolium (MTT) assay. On the first day, the cells of ACHN, A498, and HK-2 were seeded into a 96-well plate at 5×10^3^ cells/well. On the second day, various concentrations of MET and JS-K were added to the wells. Then, the cells of each well were added 20 μL of MTT (3-(4, 5-dimethylthiazol-2-yl)-2, 5-diphenyl tetrazolium bromide) (Sigma Aldrich, St. Louis, MO, USA) and incubated at 37°C for 4 h. Subsequently, the medium of each well was replaced by DMSO (150 μL) to dissolve the sediment and were shaken for 10 min in the dark. The absorbance of the solution was detected at 492 nm using a Multiskan Ascent microplate photometer (EnSpire 2300 Multilabel Reader, PE, USA).

### Cytotoxicity assay

The lactate dehydrogenase (LDH) Cytotoxicity Assay Kit (Beyotime) was used to measure the cytotoxicity of MET, JS-K, and their combination. Briefly, the cells were treated with series concentrations of MET and JS-K for 24 h after they were seeded in 96-well plates at 5×10^3^ cells/well overnight, the culture media were centrifuged at 400 × g for 5 min. The supernatants (120 μL/well) were transferred into new 96-well plates, and 60 μL of LDH detection reagent was added to each well. The plates were incubated at room temperature in the dark for 30 min, and the absorbance of the formazan was detected at 490 nm using a reader (EnSpire 2300 Multilabel Reader, PE, USA).

### Colony formation assay

ACHN and A498 cells were plated into 6-well plates and treated with various concentrations MET and JS-K for 24 h. The cells were washed with PBS and trypsinized (using 0.25% trypsin). Then, 2,000 viable cells were plated into new 6-well plates. ACHN cells were cultured at 37°C for an additional 14 days while A498 were cultured for an additional 10 days. The cells were fixed using 4% paraformaldehyde, and then stained with crystal violet (Beyotime Institute of Biotechnology, Shanghai, China). The macroscopic colonies (>50 cells) were photographed using a digital camera, and the number of colonies formed was counted.

### Apoptosis assay

The fluorescein isothiocyanate (FITC)-labeled Annexin V Apoptosis Detection kit (BD Biosciences, Franklin Lakes, NJ, USA) was used to quantify apoptotic cells. ACHN and A498 cells were collected after treatment with various concentrations of MET and JS-K for 24 h, then washed with PBS, and resuspended in binding buffer. Subsequently, cells were stained with FITC-labeled annexin V and propidium iodide for 15 min at room temperature in dark. The flow cytometry BD FACSDiva 6.1 software was used to analyze the stained cells within 1 h. The excitation wavelength of the fluorophore was 488nm, the emission wavelength of the fluorophore was PI: 564-606nm, FITC: 515-545nm.

### Intracellular ROS detection

The ROS Assay Kit (Beyotime, Beijing, China) was used to detect ROS production in ACHN and A498 cells. After being treated with various concentrations of MET and JS-K for 24 h, the ACHN and A498 cells were collected and resuspended with a serum-free medium that contained DCFH-DA (10 µM). After incubation for 20 min at 37℃ in dark, cells were analyzed by flow cytometry with excitation at 488nm and emission at 525nm. The results were analyzed using BD FACSDiva 6.1 software.

### Measurement of mitochondrial membrane potential (∆Ψm)

JC-1 Mitochondrial Membrane Potential Assay Kit (Beyotime, Shanghai, China) was used to measure mitochondrial membrane potential in ACHN and A498 cells. Briefly, ACHN and A498 cells were plated into 6-well plates at 3×10^5^ cells/well and cultivated overnight. Subsequently, MET and JS-K of series concentrations were taken to treat these cells for 24 h. The cells were harvested, washed twice with PBS, and then resuspended in 0.5 mL of complete medium containing 10 μg/mL JC-1 at 37°C for 20 min. Finally, using the BD FACSDiva 6.1 software to analyze the cells after collected.

### Western blot analysis

The A498 and ACHN cells treated with series of concentrations of MET and JS-K for 24 h were lysed in radioimmunoprecipitation assay (RIPA) buffer (Beyotime, Shanghai, China) supplemented with 1 mM phenylmethanesulfonyl fluoride (PMSF) (Beyotime, Shanghai, China) to extract the whole cell protein at 4°C. However, Cyto-C in the cytoplasm was extracted using Cell Mitochondria Isolation Kit (Beyotime, Shanghai, China). About 30 μg of protein were separated on a 10% sodium dodecyl sulfate-polyacrylamide gel and then blotted onto polyvinylidene fluoride (PVDF) membranes (Millipore, Bedford, MA, USA) at 4˚C. Subsequently, 5% non-fat milk in tris-buffered saline and 1% Tween 20 (TBST) were used to block the PVDF membranes at room temperature for 1 h and treated with specific primary antibodies with the following dilutions: Bak, 1:1,000; caspase-3, 1:1,000; caspase-9, 1:1,000; Bcl-2, 1:1,000; Bax, 1:1,000; Cyto-C, 1:1,000; PCNA, 1:1,000; Rad51, 1:1,000; γH2AX, 1:1,000; and GAPDH, 1:100,000 overnight at 4°C. Blots were washed thrice with TBST and the HRP-conjugated IgG-secondary antibody (dilution, 1:10,000) was added and the membranes were incubated at room temperature for 2 h. The protein bands were detected using an enhanced chemiluminescence kit (EMD Millipore, Billerica, MA, USA) with Tanon 5200 chemiluminescent imaging system (Shanghai, China).

### Immunofluorescence analysis

After treating ACHN and A498 cells grown on chamber slides with various concentrations of MET and JS-K for 48 h, the cells were washed with PBS, and fixed with 4% paraformaldehyde at room temperature for 30 min. The cells were permeabilized in 0.2% Triton X-100 for 30 min after being washed thrice with PBS, and then blocked for 1 h in PBS containing 1% BSA (Solarbio, Beijing, China). 100 µL of 1% BSA containing 1:100 diluted anti-γH2AX polyclonal Ab (CST) was used to suspend the cells overnight at 4 ℃. Next day, the cells were incubated in 100 µL of 1:100 diluted Alexa Fluor 488-conjugated anti-rabbit IgG (Thermo Fischer Scientific, Carlsbad, CA, USA) for 2 h at room temperature keep away from light after being washed twice with PBS. After washing the cells thrice with PBS, the Hoechst 33342 (Sigma-Aldrich, St. Louis, MO, USA) was used to stain the cells for 3 min, and the cells were photographed using a microscope (Olympus FV3000, Tokyo, Japan).

### Statistical analysis

All the data analysis was performed using SPSS 19.0 software (SPSS, Inc., Chicago, IL, USA). One-way ANOVA test followed by a post-hoc test-LSD was used to determine the significance. *p<0.05, **p<0.01, and ***p<0.001 were considered statistically significant. All experiments were performed in triplicate. Data were expressed as the means ± standard deviation.

## Results

### MET inhibits RCC cell growth

**Figure 1:**
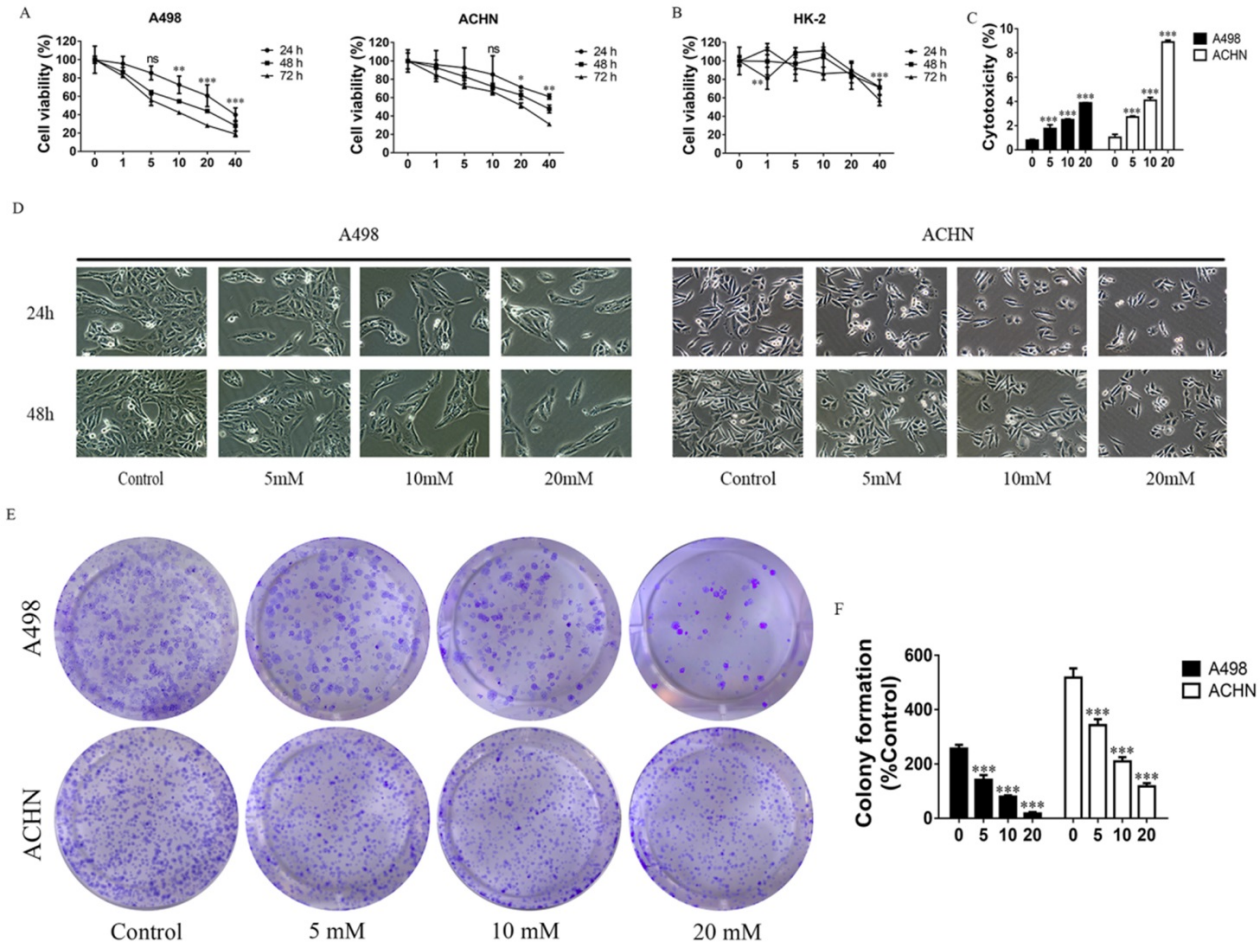
Effects of MET on RCC cell growth. Cells were treated with 1, 5, 10, 20, and 40 mM MET for 24, 48, and 72 h. The MTT assay was performed to determine cell viability. **(A)** MET inhibited the proliferation of RCC cell lines ACHN and A498 in a concentration- and time-dependent manner. **(B)** MET did not inhibit cell proliferation in normal renal cell HK-2. **(C)** Cytotoxicity of MET was investigated by LDH Cytotoxicity Assay Kit.** (D)** RCC cell lines ACHN and A498 were treated with various concentrations of MET (5, 10, and 20 mM) for 24 and 48 h. Morphologic changes (magnification, x100) in RCC cells were examined by phase contrast microscopy.** (E and F)** Cells were treated with MET (5, 10, and 20 mM) for 24 h. Colony formations were stained and counted. The data are presented as the mean ± SD for three experiments. *P < 0.05, **P < 0.01, and ***P < 0.001 compared with untreated cells.

The proliferation of cells was detected by MTT assay. MET (1, 5, 10, 20, and 40 mM) were used to treat the RCC cell lines - ACHN and A498, and HK-2 (a normal renal cell line) for 24, 48, and 72 h. Data showed that MET inhibited proliferation of ACHN and A498 cell in a concentration- and time-dependent manner (Figure 1A), whereas MET did not significantly affect the viability of HK-2 cells up to 20 mM concentrations (Figure 1B). The cytotoxicity of MET was investigated by LDH Cytotoxicity Assay Kit, and the data showed that treatment with MET for 24 h increased cytotoxicity (Figure 1C) in RCC cells in a concentration-dependent manner. Morphologic changes in renal carcinoma cells were observed through phase contrast microscopy. After treatment with MET (0, 5, 10, and 20 mM) for 24 and 48 h, the degree of confluence in A498 and ACHN were significantly decreased in conjunction with marked morphologic changes, that is, cells appeared as atypical shape, shrunken and rounded, and detached from the culture dish (Figure 1D). A colony formation assay was used to test the effect of MET on tumor cell colony formation. Compared to the control group, the colony counts of RCC cells treated with MET (0, 5, 10, and 20 mM) for 24 h were significantly decreased (Figure 1E and F). Collectively, these data suggested that MET evidently inhibited the cell growth of the RCC cell lines ACHN and A498 but only minimally affected the nonmalignant HK-2 cells.

### MET promotes RCC cell apoptosis by increasing ROS production and activating the mitochondria-dependent apoptotic pathway

**Figure 2:**
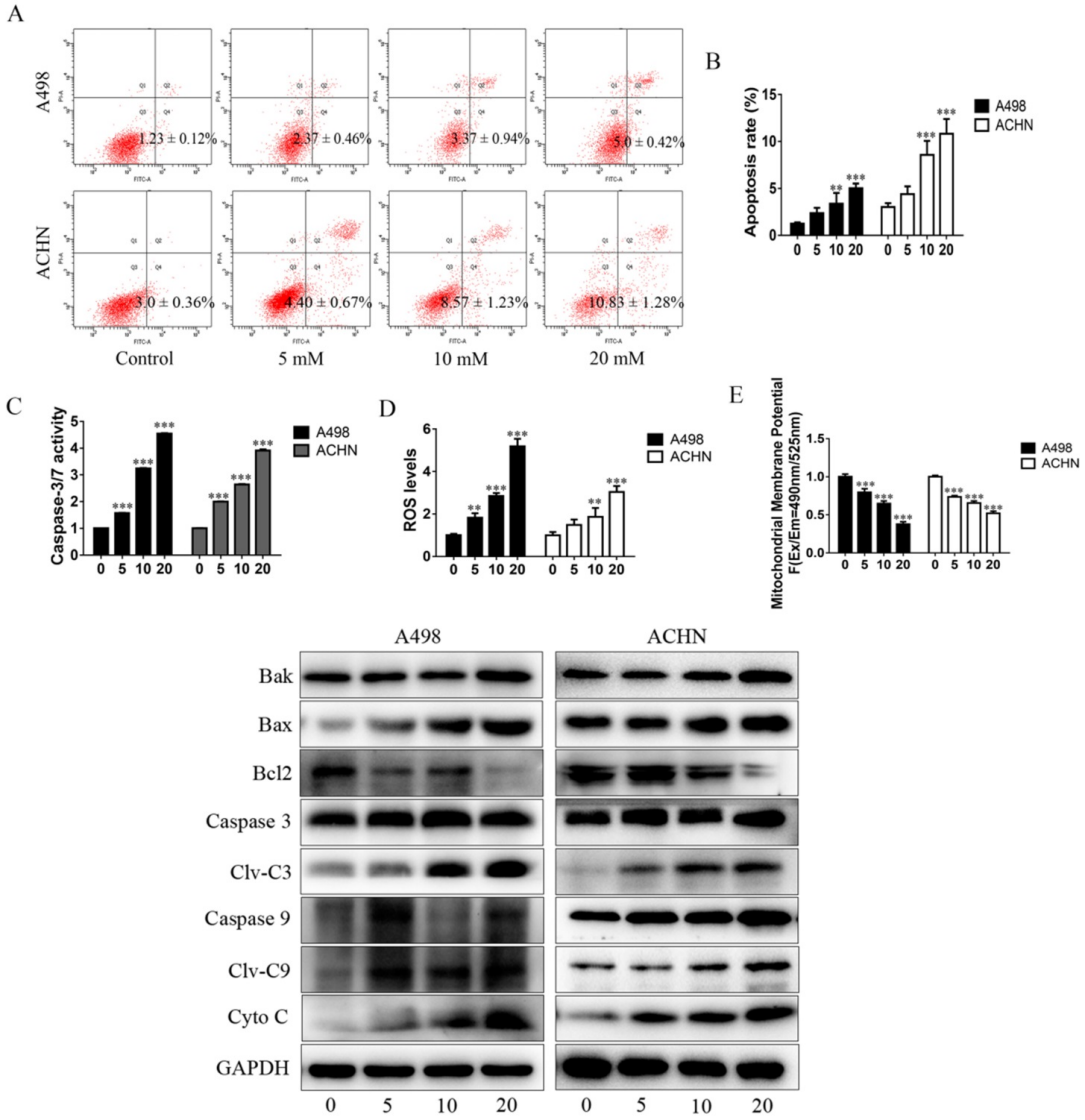
MET promoted apoptosis of RCC cells by increasing ROS production and mitochondria-dependent apoptotic pathway. RCC cell lines ACHN and A498 were exposed to various concentrations of MET (5, 10, and 20 mM) for 24 h, and the apoptosis rates **(A and B)**, caspase-3/7 activity **(C)**, intracellular level of total ROS **(D)**, and mitochondrial membrane potential **(E)** were detected. **(F)** Expression of apoptotic proteins, such as caspase-3, caspase-9, Bak, Bax, Cyto C, and Bcl-2, were detected by Western blot. The data are presented as the mean ± SD for at least three independent experiments. **P < 0.01 and ***P < 0.001 compared with untreated cells.

The apoptosis-inducing effect of MET on RCC cells was evaluated by flow cytometry. The RCC cells were treated with MET (5, 10, and 20 mM) for 24 h to assess whether it induces apoptosis in a concentration-dependent manner (Figure 2A and B). The dihydroethidium and JC-1 fluorescent methods were used to measure the effect of MET on ROS production and mitochondrial transmembrane potential (Δψm). The RCC cells treated with a series of concentrations of MET for 24 h were examined for total ROS levels and mitochondrial transmembrane potential. A significant increase in the total ROS (Figure 2D) and a decrease in the mitochondrial transmembrane potential (Figure 2E) were observed in the RCC cells ACHN and A498 in a concentration- dependent manner. These data suggested that MET induces ROS production and promotes mitochondrial dysfunction and mitochondria-dependent apoptosis.

The activity of caspase-3/7 and the expression of apoptotic proteins were detected in the RCC cells following treatment with MET for 24 h. The results showed that MET increases the activity of caspase-3/7 (Figure 2C). The pro-apoptotic proteins such as cleaved caspase-3, cleaved caspase-9, Bak, Bax, and Cyto-C were up-regulated, whereas the anti-apoptotic protein (Bcl-2) was down-regulated in a concentration-dependent manner in MET-treated cells compared with the controls (Figure 2F).

### MET + JS-K suppressed the RCC cell growth

**Figure 3:**
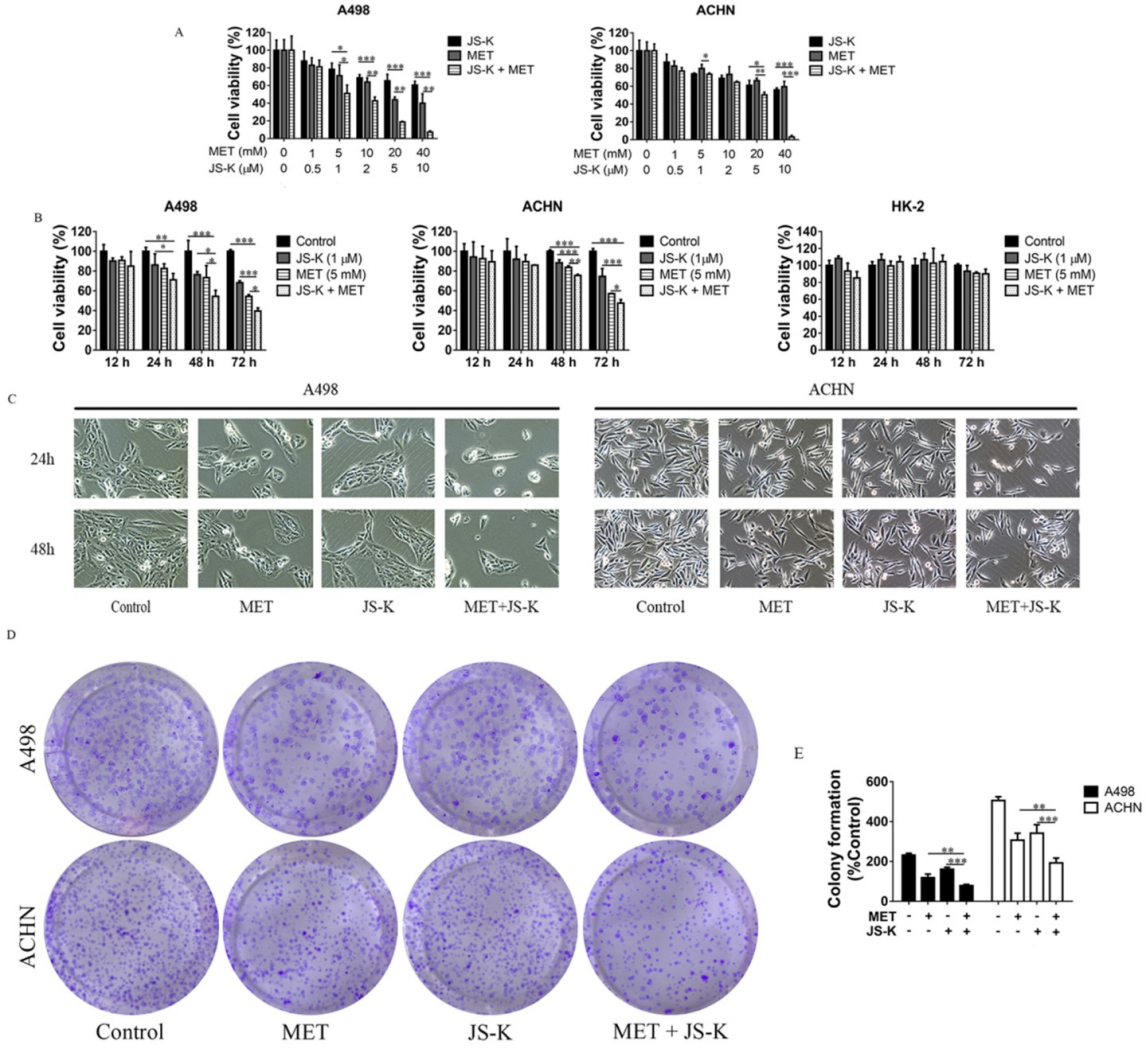
MET + JS-K suppressed RCC cell growth. **(A)** RCC cells were exposed to MET, JS-K, or both for 48 h. The MTT assay showed that cell growth was remarkably inhibited by MET and JS-K in a concentration-dependent manner. **(B)** MET + JS-K suppressed RCC cell proliferation in a time-dependent manner, but the human normal renal HK-2 cells were insensitive to either MET or JS-K. **(C)** Changes in the morphology of the ACHN and A498 cells treated with MET (5 mM) and JS-K (1 µM). **(D and E)** MET and JS-K suppressed cell colony formation. The data are presented as the mean ± SD for at least three experiments. *P < 0.05, **P < 0.01, and ***P < 0.001 compared with untreated cells.

The RCC cells were exposed to MET (1, 5, 10, and 20 mM), JS-K (0.5, 1, 2, 5, and 10 μM), or both for 48 h. The MTT assay showed that cell viability was significantly inhibited by MET and JS-K and was significantly decreased when cells were treated using MET + JS-K in a concentration-dependent manner (Figure 3A). When the RCC and HK-2 cells were treated with MET (5 mM) and JS-K (1 μM) for 12, 24, 48, and 72 h, MET + JS-K suppressed RCC cell viability in a time-dependent manner but did not affect HK-2 cells (Figure 3B). Specifically, the RCC cells treated with MET (5 mM) and JSK (1 µM), decreased cell number and changed cell morphology (Figure 3C). Moreover, MET + JS-K enhanced the effect. Figure 3D and E demonstrate that MET or JS-K alone suppressed cell colony formation, but MET + JS-K significantly suppressed RCC cell colony formations compared with the single drug.

### MET + JS-K promotes apoptosis in the RCC cells by increasing ROS production and activating mitochondria-dependent apoptotic pathway

**Figure 4:**
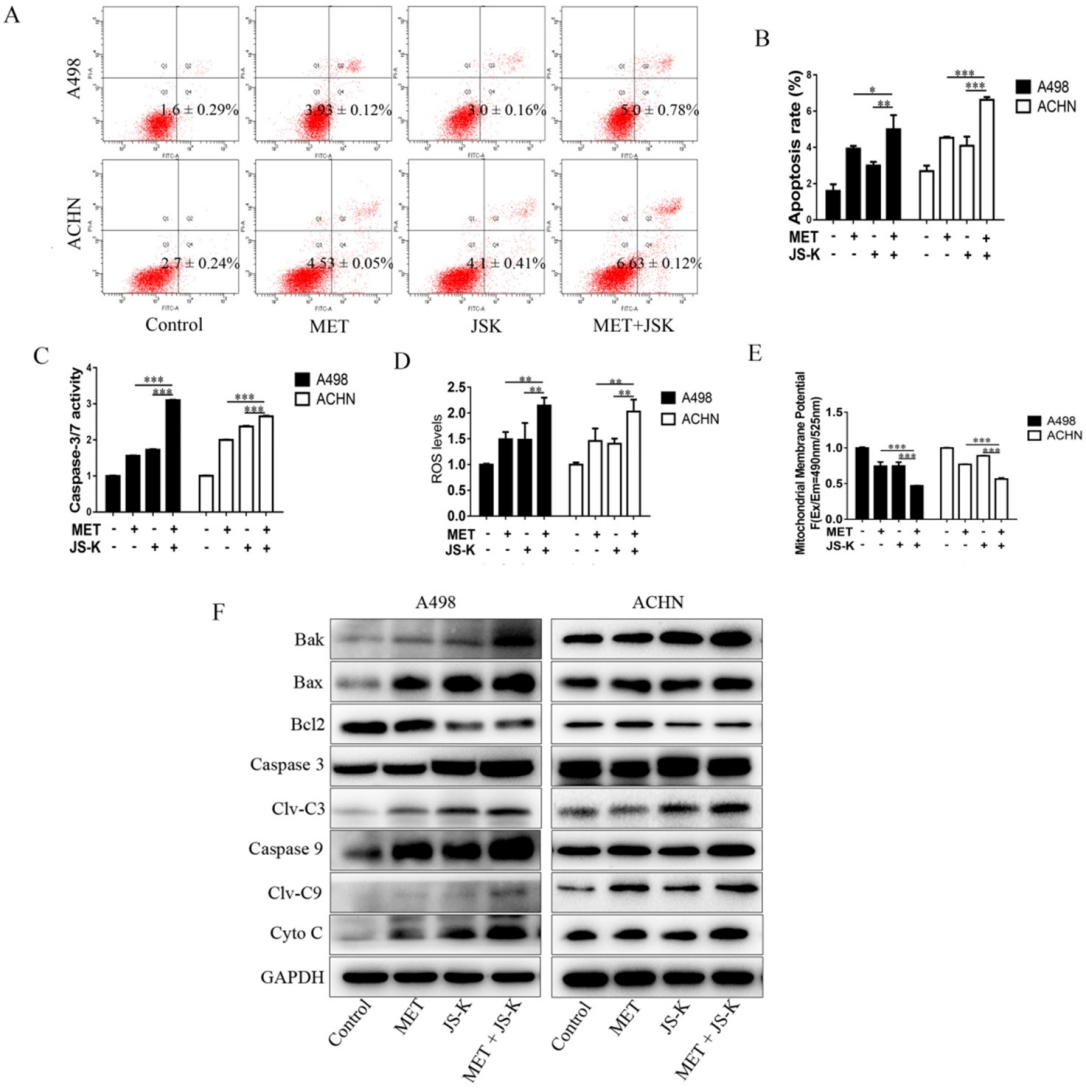
MET + JS-K promotes apoptosis in RCC cells by increasing ROS production and mitochondria-dependent apoptotic pathway. RCC cells were exposed to MET (5 mM), JS-K (1 µM), or their combination for 24 h. **(A and B)** Cell apoptosis rates. **(C)** Activity of caspase-3/7. **(D)** ROS production. **(E)** Mitochondrial membrane potential was tested. **(F)** Protein levels of Bax, Bak, Cyto C, cleaved caspase-3, and cleaved caspase-9 were detected by Western blot. The data are presented as the mean ± SD for at least three independent experiments. *P < 0.05, **P < 0.01, and ***P < 0.001 compared with untreated cells.

The RCC cells were exposed to MET (5 mM), JS-K (1 μM), or MET+JS-K for 24 h, and their apoptotic effect was investigated. The result showed that cell apoptosis is significantly promoted by MET + JS-K compared with MET or JS-K alone (Figure 4A and B). The effects of MET and JS-K on ROS production were evaluated. The ROS production in RCC cells treated with MET + JS-K was significantly increased (Figure 4D). Besides, the mitochondrial membrane potential was significantly decreased in the RCC cells treated with MET + JS-K compared with MET or JS-K treatments alone (Figure 4E).

The cells treated with MET + JS-K had a higher activity of caspase-3/7 (Figure 4C) than cells treated with MET or JS-K alone. The protein levels of Bax, Bak, Cyto-C, cleaved caspase-3, and cleaved caspase-9 were up-regulated in response to MET + JS-K, whereas the Bcl-2 levels were down-regulated (Figure 4F).

### Anticancer activity of MET + JS-K on the RCC cells was reversed by NAC and exacerbated by GSSG

**Figure 5:**
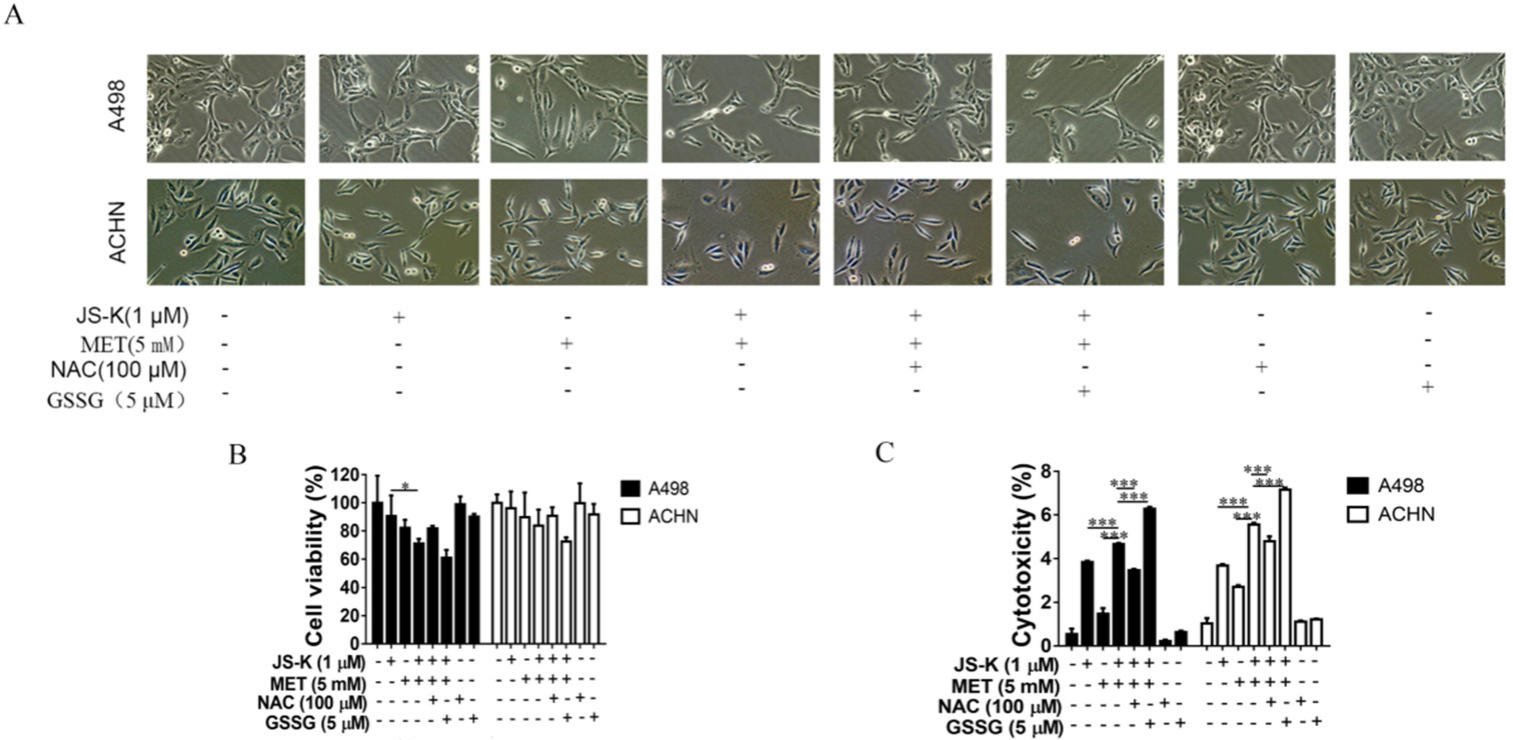
Anticancer activity of MET + JS-K on RCC cells was reversed by NAC and exacerbated by GSSG. The ACHN and A498 cells were cultured with 100 μM NAC or 5 μM GSSG for 24 h and then treated with or without MET (5 mM) + JS-K (1 µM). **(A)** Cells were visualized by microscopy (100×). **(B)** Cell survival was detected by MTT assay. **(C)** Cytotoxicity was investigated using an LDH Cytotoxicity Assay Kit. The data are presented as the mean ± SD for three experiments. *P < 0.05, **P < 0.01, and ***P < 0.001 compared with untreated cells.

**Figure 6:**
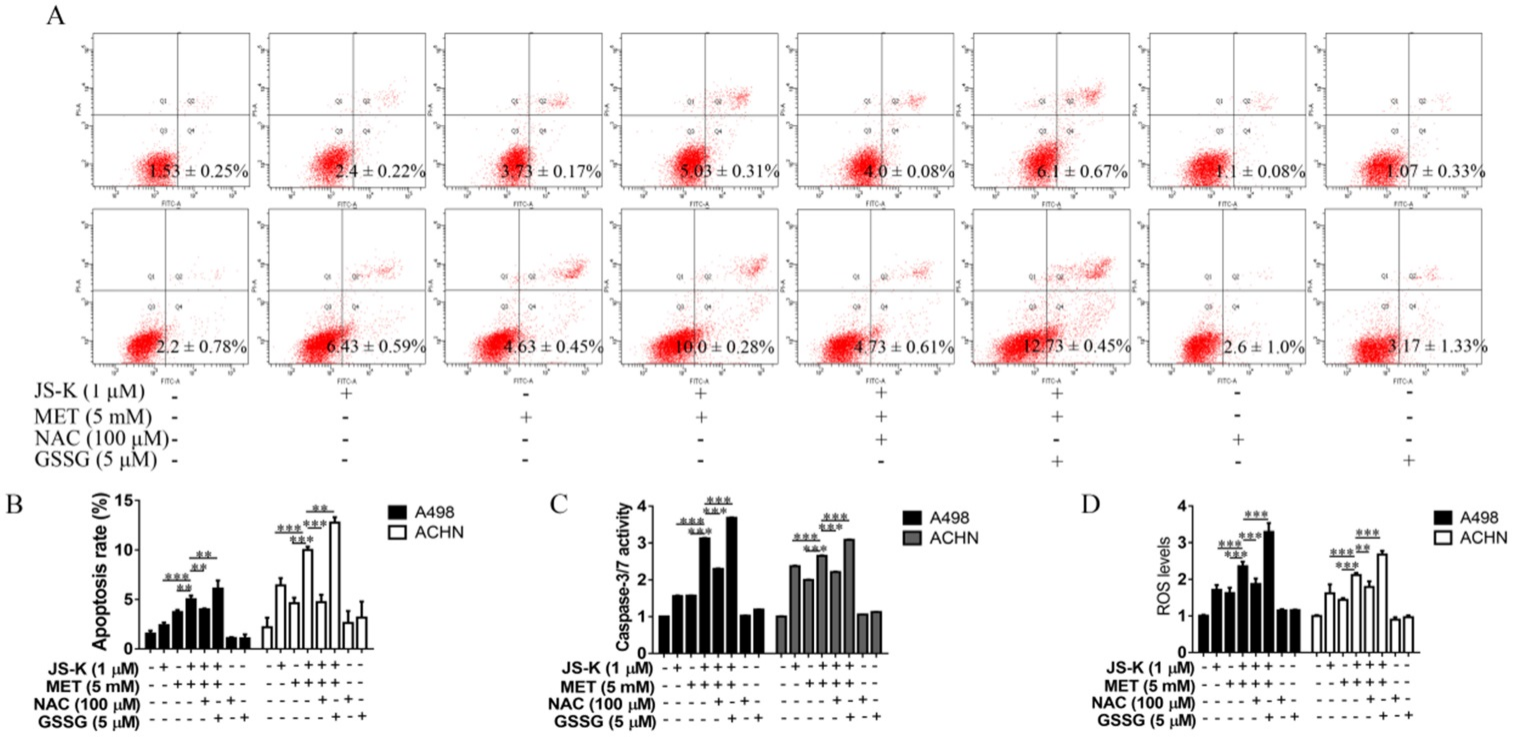
Effects of NAC and GSSG on MET and JS-K-induced cell apoptosis. The ACHN and A498 cells were cultured with 100 μM NAC or 5 μM GSSG for 24 h and then treated with or without MET (5 mM) + JS-K (1 µM). **(A and B)** Apoptosis of cells was analyzed by FITC-annexin V/PI staining. Cumulative results were presented. **(C)** Caspase-3/7 activity of the ACHN and A498 cells. **(D)** Accumulation of intracellular ROS in RCC cells. The data are presented as the mean ± SD for three independent experiments. **P < 0.01 and ***P < 0.001 compared with untreated cells.

The effects of ROS on MET and JS-K in the RCC cell proliferation suppression and apoptosis were explored using RCC cells ACHN and A498 treating with MET (5 mM) and JS-K (1 μM) in the presence or absence of the antioxidant N-acetylcysteine (NAC; 100 µM) or pro-oxidant oxidized glutathione (GSSG; 5 µM) for 24 h. NAC blocked MET+JS-K-induced cell growth suppression (Figure 5A and B), cell cytotoxicity (Figure 5C), apoptosis (Figure 6A and B), caspase-3/7 activity (Figure 6C), and ROS levels (Figure 6D). In contrast, treatment with GSSG augmented the MET+JS-K-induced cell growth inhibition (Figure 5A and B), cytotoxicity (Figure 5C), apoptosis (Figure 6A and B), caspase-3/7 activity (Figure 6C), and ROS levels (Figure 6D). These findings suggest that MET + JS-K inhibits cells growth and promotes the apoptosis of the RCC cells through ROS production.

### MET and JS-K increase DNA breaks and inhibit the expression of PCNA and Rad51

**Figure 7:**
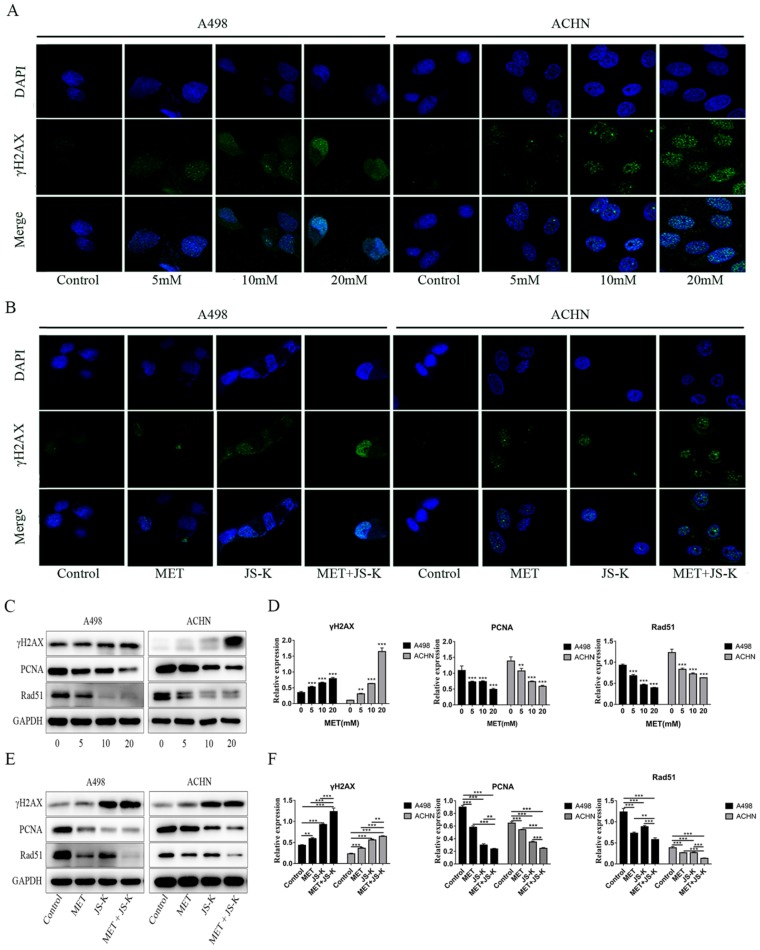
MET and JS-K increased DNA breaks and restrained the expression of PCNA and Rad51. The γH2AX foci in the RCC cells were tested by immunofluorescence analysis. **(A)** MET increased γH2AX foci to induce DNA breaks in a concentration-dependent manner in the RCC cells ACHN and A498. **(C)** MET + JS-K dramatically increased DNA breaks compared with MET or JS-K alone. **(B and E)** Effect of MET alone or MET + JS-K (1 µM) on the expression of PCNA, and Rad51 and γH2AX were detected through Western blot assay. (D, F) All three proteins were quantified using Image J software and normalized against GAPDH.

The immunofluorescence analysis was used to detect γH2AX foci in the RCC cells. The concentrations of 5, 10, and 20 mM MET alone or MET (5 mM) + JS-K (1 µM) were used to treat RCC cells for 48 h. Figure 7A depicts the MET-induced DNA breaks in a concentration-dependent manner in the RCC cells ACHN and A498. In addition, MET + JS-K dramatically increased the DNA breaks compared with MET or JS-K alone (Figure 7C). We also detected the effect of MET alone or MET + JS-K on the expression of PCNA, Rad51, and γH2AX-the three important proteins involved in the DNA break and repair. Figure 7B displays that MET extensively increased the expression of γH2AX and reduced the expression of PCNA and Rad51. Similar effects were seen after treatment with MET + JS-K compared with MET alone (Figure 7D). These data showed that MET increases DNA breaks in the RCC cells through modulation of the cellular DNA repair pathway, and the DNA damaging effects of MET are augmented when combined with JS-K.

## Discussion

At present, surgery, chemotherapy, radiotherapy, immunotherapy, and molecular targeted therapy are the standard treatments for patients with RCC. However, the therapeutic effect of immunotherapy and molecular targeted therapy are limited given their high toxicity profile and cost 26, 27. Moreover, advanced RCC is highly chemo-resistant 5, 6. Our goal is to identify an effective and less toxic treatment with few side effects for patients with RCC. MET is an appropriate drug with considerable anti-tumor effects and fewer side-effects. In the present study, MET inhibits proliferation and promotes apoptosis in the RCC cells ACHN and A498 in a concentration- and time-dependent manner, whereas these identical concentrations do not elicit cytotoxicity in normal renal tubular epithelial cells.

Apoptosis is a kind of programmed cell death produced by many physiological and pathological conditions and deregulated apoptotic signaling is considered to play a crucial role in the development and progression of cancer 28, 29. Two ways by which cells can initiate apoptosis are mitochondrial and death receptor pathways 30. Many chemical and physiological stimuli that can induce apoptosis are known to provoke oxidative stress by generating excess ROS, which suggests that abnormal ROS generation is closely associated with apoptosis 31. Abnormal ROS generation plays a vital role in mitochondria-dependent apoptosis, known as intrinsic apoptosis. As signaling molecules, ROS participates in multiple cellular functions, including cell proliferation, cell cycle progression, invasion, migration, and apoptosis in cancer 32. ROS generations induce damage and depolarization of the mitochondrial membrane and accordingly decrease mitochondrial membrane potential and increase the levels of other pro-apoptotic molecules in the cells 33. Considerable research has indicated that a high level of ROS is required for initiating apoptotic responses, which is often one of the prominent mechanisms of several anticancer drugs. Therefore, we examined the induction of ROS, alterations in mitochondrial membrane potential, and the onset of apoptosis at various concentrations of MET on the RCC cells ACHN and A498. Our findings provide direct evidence that MET enhances ROS generation, induces damage, and depolarizes the mitochondrial membrane in the RCC cells. These factors are responsible for the pro-apoptotic effects of MET on the RCC cells. Figure 2 displays that MET induces apoptosis and ROS generation; decreases mitochondrial membrane potential; up-regulates protein levels of Bax, Bak, Cyto-C, cleaved caspase-3, and cleaved caspase-9; and down-regulates Bcl-2 in the ACHN and A498 cells, thus indicating that MET exerts anticancer activity by increasing ROS production and inducing mitochondria-dependent apoptosis. In addition, as ROS have a short-half life, highly reactivity, and an electronegative oxygen, they can induce DNA damage and affect the DNA damage response 34. Chemotherapeutic agents, such as doxorubicin and cisplatin, increase ROS levels, which contribute to their cytotoxicity by increasing DNA damage 35, 36. To evaluate whether MET induces DNA damage in ACHN and A498, the immunofluorescence analyses was used to detect γH2AX foci, an important marker for DNA damage in the cells. Our results showed that the number of γH2AX foci increased in cells treated with 5, 10, and 20 mM MET, thereby indicating that MET induces DNA damage in RCC cells ACHN and A498 in a concentration- dependent manner (Figure 7A). Analogously, the expression of the γ-H2AX protein in the RCC cells treating with MET was increased (Figure 7B). The increased cellular DNA damage might be caused by the excess ROS generation and result in subsequent apoptosis and suppression of proliferation in the RCC cells ACHN and A498. Furthermore, MET suppressed the growth of carcinoma cells by inhibiting the DNA damage repair pathway 37. To assess the role of MET in regulating cellular DNA repair, we assessed the effect of MET on the expression of PCNA and Rad51, the two markers that serve crucial functions in DNA repair. The data revealed that MET extensively restrains the expression level of PCNA and Rad51 (Figure 7B), thus suggesting that MET increases genotoxicity in RCC cells by decreasing the cellular DNA repair mechanisms.

In addition, our previous study demonstrated that adding JS-K up-regulates p53 and increases intracellular ROS accumulation to upsurge the anticancer effects of doxorubicin 38. In the study, inhibiting RCC cell growth was further potentiated when JS-K was added. This combined effect may be related to intracellular ROS accumulation because excess ROS generation is considered to sensitize tumor cells to chemotherapy drugs 39, 40. Therefore, we hypothesized that JS-K would increase the generation of ROS in RCC cells, which would subsequently sensitize them to even a low level of MET. Our study showed that MET + JS-K remarkably increases the generation of ROS, suppresses proliferation (Figure 3), and induces mitochondria- dependent apoptosis (Figure 4) and DNA breaks (Figure 7C and D) compared to treatment with MET or JS-K alone. Moreover, the enhanced cytotoxic effect by combining the agents was largely abolished by NAC and exacerbated by GSSG (Figure 5 and 6). Thus, MET + JS-K reactivated oxygen species activation, induced DNA breaks, and increased the inhibition of RCC cell growth.

In conclusion, our studies revealed that MET inhibits cell proliferation and induces apoptosis and DNA breaks in the RCC cells ACHN and A498. Moreover, treatment with MET + JS-K synergistically inhibits cell growth and induces cell apoptosis and DNA breaks in the RCC cells through ROS activation. This combination therapy could be a promising option for patients with RCC.

## Abbreviations

MET: Metformin
RCC: human renal cell carcinoma
Δψm: mitochondrial transmembrane potential
PBS: phosphate buffer saline
DMSO: dimethyl sulfoxide
NAC: N-acetylcysteine
GSSG: glutathione disulfide
HRP: Horseradish peroxidase
MTT: methyl-tetrazolium
LDH: lactate dihydrogenase
FITC: fluorescein isothiocyanate
RIPA: radio immunoprecipitation Assay
SDS-PAGE: sodium dodecyl sulfate polyacrylamide gel electrophoresis
TBST: Tris-buffered saline and 1% Tween 20
