# Metformin in combination with JS-K inhibits growth of renal cell carcinoma cells via reactive oxygen species activation and inducing DNA breaks: Erratum

**DOI:** 10.7150/jca.76980

**Published:** 2022-08-19

**Authors:** Yuwan Zhao, Qiuming Luo, Jierong Mo, Jianwei Li, Dongcai Ye, Zhixian Ao, Lixin Chen, Jianjun Liu

**Affiliations:** Laboratory of Urology, Affiliated Hospital of Guangdong Medical University, Zhanjiang, Guangdong 524001, China

Recently, we conducted an examination of our published article and found errors in Fig 5A.

In Fig 5A, the control group and NAC group's cell morphology picture of A498 were repeated. It was a mistake. When the pictures were combined, the names of the groups were not carefully checked. Also, the cell morphology images of NAC and GSSG groups of ACHN were repeated. We made these mistakes during the assembly of the images. Below is the corrected figure 5.

## Figures and Tables

**Figure 5:**
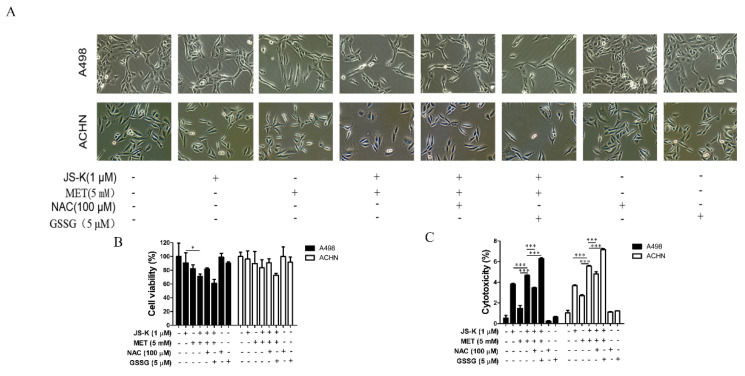
Corrected figure.

